# Kuntai Capsules Improve Premature Ovarian Failure by Regulating AMPK-Mediated Autophagy

**DOI:** 10.1007/s43032-025-01949-w

**Published:** 2025-08-11

**Authors:** Xiaomin Ye, Miao Chen, Jiajing Zhong, Haofan Chen, Xinmiao Lin

**Affiliations:** 1 Central People’s Hospital of Zhanjiang, Zhanjiang, Guangdong 524045 China; 2 Department of Obstetrics and Reproductive Health and Infertility, Central People’s Hospital of Zhanjiang, Zhanjiang, Guangdong 524045 China

**Keywords:** Kuntai capsule, Premature ovarian failure, Follicular granulosa cell, AMPK/mTOR signaling pathway, Autophagy

## Abstract

Background: Premature ovarian failure (POF) is a gynecological endocrine disorder with current treatments having limitations. Kuntai capsule (KTC), a traditional Chinese medicine formulation, is thought to be beneficial for POF, but its mechanism is unclear. Network pharmacology can help explore drug mechanisms. Methods: A POF rat model was established using cyclophosphamide (CTX). Rats received low-dose KTC (0.6 g/kg/d), high-dose KTC (1.8 g/kg/d), or dehydroepiandrosterone (DHEA, positive control). Ovarian function was evaluated via histopathology, hormone assays (ELISA), apoptosis (TUNEL/flow cytometry), autophagy markers (Western blot), and network pharmacology. Results: KTC treatment (especially high-dose) ameliorated POF in CTX-treated rats, as shown by increased ovarian weight, restored estrus cycle, and improved follicle development. The serum estradiol (E2), anti-mullerian hormone (AMH), and superoxide dismutase (SOD) levels increased, whereas the follicle-stimulating hormone (FSH), malondialdehyde (MDA), and reactive oxygen species (ROS) levels decreased following KTC treatment. KTC also alleviated ovarian cell apoptosis and autophagy, with higher-dose KTC being more effective. Network pharmacology predicted AMPK/mTOR pathway involvement. Western blot confirmed KTC activated the AMPK/mTOR signaling, downregulated autophagy markers (LC3B-II/I, Beclin1), and upregulated P62. Autophagy inhibition (via 3-MA) mirrored KTC effects, while mTOR blockade (rapamycin) reversed them. Conclusions: KTC ameliorates POF by inhibiting excessive ovarian autophagy through AMPK/mTOR pathway activation, providing a mechanistic basis for its clinical use.

**Supplementary Information:**

The online version contains supplementary material available at 10.1007/s43032-025-01949-w.

## Background

Premature ovarian failure (POF) is a gynecological endocrine disorder characterized by estrogen deficiency and elevated gonadotropin levels in women before the age of 40 years. The primary pathological changes associated with POF include reduced development of mature ovarian follicles and an increase in atretic follicles [1]. In recent years, the age of onset for POF has gradually decreased [2]. This condition not only significantly impacts women's work and daily life but also imposes considerable stress and burden on patients' families and society at large. Currently, treatment for POF primarily focuses on symptomatic management, including hormone replacement therapy (HRT), ovulation induction, and immunotherapy. However, these treatments may elevate the risk of ovarian cancer, cervical cancer, and other malignancies to some extent [3, 4]. Consequently, there is a pressing need for safe and effective pharmacological interventions for the treatment of POF.

According to traditional Chinese medicine, POF may be attributed to several factors, including kidney essence deficiency, liver blood deficiency, and hyperactivity of heart fire [5]. Kuntai capsule (KTC) is a traditional Chinese medicine formulation composed of cooked rehmannia, Coptis, scutellaria, paeony, ejiao, and poria [6]. This preparation is believed to nourish Yin, clear heat, calm the nerves, and alleviate irritability, making it suitable for patients experiencing ovarian function decline due to Yin deficiency and excess fire [7]. Research indicates that KTC can improve various gynecological conditions, including POF, polycystic ovary syndrome (PCOS), climacteric syndrome, and endometriosis [7–9]. However, the precise mechanism of action of KTC remains unclear. Some studies have reported that KTC alleviates symptoms of POF by activating the FOXO3/SIRT5 signaling pathway in mice [10]. Additionally, KTC has been shown to enhance ovarian function in POF rats by upregulating the expression of growth differentiation factor 9 (GDF-9), light chain 3 A-II, and Beclin 1 [11].

Most drugs have multiple therapeutic targets and are implicated in various diseases through different mechanisms [12]. Network pharmacology analysis allows for the examination of the pharmacological effects of drugs from multiple perspectives, exploring them across different biological layers including molecules, cells, and tissues [13]. This analytical approach predicts the active ingredients of drugs and their therapeutic targets at a systemic level, thereby establishing a complex network linking drug components to disease targets [14]. Bioinformatics analysis was employed to import the overlapping genes of the two types into the STRING database, facilitating the construction of a protein–protein interaction (PPI) network. Subsequently, corresponding software was utilized for visualization, where nodes in the network were organized according to a specified algorithm, revealing the relationships between drugs and targets pertinent to the study of core genes [15]. Network pharmacology analysis has been extensively applied to investigate the biological mechanisms underlying various drug formulations and components [16–18].

This study aims to validate the feasibility of KTC in treating POF and employs network pharmacology analysis to predict the primary active components of KTC, along with its key targets and potential signaling pathways in the context of POF, thereby providing a theoretical foundation for the treatment of this condition using KTC.

## Methods

### In Vivo Experiment

#### Construction of POF Rat Model and KTC Treatment

This experiment was approved by the Experimental Animal Ethics Committee of Guangzhou Miers Biotechnology Co., LTD. (IACUC-MIS20230069). SD female rats (6–8 weeks old, 200 ± 5 g) were purchased from Guangdong Medical Laboratory Animal Center. The POF rat model was constructed according to the previous studies [19, 20]. Cyclophosphamide (CTX, Cat.HY-17420, MCE, USA) was injected intraperitoneally for 14 days with an injection dose of 50 mg/kg on the first day and 8 mg/kg on the subsequent day. KTC (Guiyang Xintian Pharmaceutical, China) suspension (2.0 mL) was intragastically administered with doses of 0.6 g/kg/d for the low dose group and 1.8 g/kg/d for the high dose group. Dehydroepiandrosterone (DHEA, Cat.HY-14650, MCE, USA) was used as a positive control and was intragastically administered with a dose of 13.5 mg/kg. The rats were weighed daily and their estrus cycles were recorded. After the experiment, the rats were euthanized, and the ovaries were removed and weighed immediately.

#### Autophagy Modulation Experiments

To elucidate the role of AMPK-mTOR signaling in KTC-mediated POF alleviation, autophagy inhibitor 3-Methyladenine (3-MA, Cat.HY-19312, MCE, USA) and mTOR-specific inhibitor Rapamycin (RAPA, Cat.HY-10219, MCE, USA) were administered to KTC-treated POF rats. The inhibitor intervention groups were designed as follows: KTC + 3-MA group (rats received intraperitoneal injections of 15 mg/kg 3-MA one hour prior to KTC-H (1.8 g/kg/d) intragastric administration twice a week), KTC + RAPA group (rats received intraperitoneal injections of 2 mg/kg RAPA one hour prior to KTC-H (1.8 g/kg/d) intragastric administration per day for two weeks). Inhibitor dosing regimens were based on established protocols for ovarian autophagy modulation [21, 22]. All inhibitors were dissolved in dimethyl sulfoxide (DMSO) and diluted with saline (final DMSO concentration < 0.1%). Control groups received vehicle solution (0.1% DMSO in saline) at equivalent volumes. The intervention lasted 14 days concurrent with KTC treatment.

### Histopathology

Fresh ovarian tissue was removed and fixed in 4% paraformaldehyde solution (Cat.P0099, Beyotime, China). After paraffin (Cat.39601095, Leica Biosystems, Germany) embedding, the tissue was sectioned to 4-μm-thick slices, hematoxylin and eosin (H&E, Cat.C0105S, Beyotime, China) staining were used to observe the ovary morphology and the number and morphology of follicles at all development stages. Follicles were classified and counted by two independent blinded observers as described [23]. Every 10th section was chosen to analyze and the raw count was multipled by ten to estimate the total number of follicles. Follicles without clear oocyte nuclei were excluded. Primordial follicles are characterized by an oocyte surrounded by one layer of flattened granulosa cells. Primary follicles have one to two complete layers of cuboidal granulosa cells surrounding the oocyte. Secondary follicles have more than two layers of cuboidal granulosa cells. Mature follicles have multiple layers of cuboidal granulosa cells with a cumulus oophorus and antral spaces.

For immunohistochemistry, paraffin sections were immersed in xylene (Cat.B83606, Sigma-Aldrich, USA) for dewaxing, and then immersed in gradient ethanol solution (Cat.459828, Sigma-Aldrich, USA) to remove xylene. To inactivate endogenous peroxidase, an appropriate amount of peroxidase blocker (Cat. P0100A, Beyotime, China) was employed. The sections were soaked in citric acid buffers (Cat.P0083, Beyotime, China) to heat repair the antigens. The antibody was added and incubated at 4℃ overnight. On the second day, the second antibody was added for incubation, hematoxylin was re-stained after DAB (Cat.P0202, Beyotime, China) color development, and 1% hydrochloric acid alcohol was returned to blue. Primary antibodies were as follows: Rabbit polyclonal anti-BMP15 (Cat.18982–1-AP, Proteintech, USA, 1:200), Rabbit polyclonal anti-BMP7 (Cat.CL488-12,221, Proteintech, USA, 1:200), Rabbit polyclonal anti-P16 (Cat.10883–1-AP, Proteintech, USA, 1:200), Rabbit monoclonal anti-γH2AX (Cat.ab229914, Abcam, UK, 1:200).

### ELISA Analysis

Serum samples were collected after centrifuging blood at 3000 rpm for 10 min. The ELISA kits used were as listed: Rat estradiol (E2) ELISA kit (Cat.KT-59814, Kamiya, USA), Rat anti-Mullerian hormone (AMH) ELISA kit (Cat.CSB-E11162r, Cusabio, China), Rat follicle-stimulating hormone (FSH) ELISA Kit (Cat.CSB-E06869r, Cusabio, China), Rat Malondialdehyde (MDA) ELISA Kit (Cat.AE20805R, A&E bio, China), Rat reactive oxygen species (ROS) ELISA Kit (Cat.J23513, GILED, Wuhan, China), and Rat Super Oxidase Dimutase (SOD) ELISA Kit (Cat.JN4055, Jining Bio, China).

The analysis was conducted according to the manufacturers’ instructions. In brief, antibody working solution and avidin-peroxidase (ABC) working solution were prepared, and 100 µl of standard product was added. The enzyme label plate was covered with sealing plate membrane and reacted at 37℃ for 90 min. The liquid in the enzyme label plate was removed and 100 µl of biotin anti-antibody working solution was added to each well, and washed with buffer solution for 3 times after incubation. OD values were measured at 450 nm by ELx800 Absorbance Microplate Reader (Bio-Tek, USA).

### Western Blot Analysis

The total protein was extracted with RIPA lysate (Cat. P0013C, Beyotime, China), separated with sodium dodecyl sulfate polyacrylamide gel (SDS-PAGE), and then transferred to polyvinylidene fluoride (PVDF) membrane (Cat.IPVH00010, Millipore, USA) and sealed with 5% skim milk powder solution. The film was incubated with the corresponding primary antibody at 4℃ overnight, and after cleaning with PBS, the film was incubated with the corresponding secondary antibody at room temperature for 2 h, and the ECL (Cat.P0018S, Beyotime, China) method was developed. The signal was detected with the iBright 1500 Image system (Thermo Fisher Scientific, Pittsburgh PA). Semi-quantitative analysis was performed on the western blot bands and intensities of the bands were quantified using image J. Primary antibodies were used as Table 1.

**Table 1:** Antibodies for Western blot analysis

Name	Catalog No	Dilution Ratio	Molecular Weight (kDa)
Rabbit polyclonal anti-AMH	14,461–1-AP, Proteintech	1:5000	59
Rabbit monoclonal anti-BMP15	ab108413, Abcam	1:1000	45
Rabbit monoclonal anti-BMP7	ab129156, Abcam	1:1000	49
Rabbit polyclonal anti-GDF 9	ab93892, Abcam	1:1000	51
Rabbit monoclonal anti-VEGFA	ab214424, Abcam	1:1000	40
Rabbit polyclonal anti-Histone H2A.X	10,856–1-AP, Proteintech	1:5000	15
Rabbit monoclonal anti-β-Galactosidase (E2U2I)	#27,198, Cell Signaling Technology	1:1000	65
Rabbit polyclonal anti-AMPK Alpha	10,929–2-AP, Proteintech	1:5000	64
Rabbit monoclonal anti-AMPK alpha 1 (phospho T183) + AMPK alpha 2 (phospho T172)	ab133448, Abcam	1:1000	64
Rabbit monoclonal anti-mTOR	ab134903, Abcam	1:1000	289
Rabbit monoclonal anti-mTOR (phospho S2448)	ab109268, Abcam	1:1000	289
Mouse monoclonal anti-GAPDH	ab8245, Abcam	1:2000	36
Goat Anti-Mouse IgG/HRP	SE131, Solab	1:2000	
Goat Anti-Rabbit IgG H&L (HRP)	ab6721, Abcam	1:2000	

### Apoptosis Detection

The TUNEL Assay Kit (Cat.ab206386, Abcam, UK) was used to detect apoptosis. In brief, soak the slices in xylene and ethanol solutions of different concentrations for dewaxing hydration, rinse ddH_2_O, add protease K (Cat.P1005, Beyotime, China) for repair, and inactivate endogenous peroxidase with 3% H_2_O_2_. It was incubated in the working liquid labeled by HRP at 37℃ and the color was developed by DAB method. Hematoxylin was re-dyed, 1% hydrochloric acid alcohol was differentiated into anti-blue, ddH_2_O was washed and dried naturally.

For flow cytometry assay, the ovarian samples were prepared into single-cell suspension, then they were stained according to the manufacturer’s instruction of the Annexin V-FITC Apoptosis Detection Kit (Cat.CA1020, Solarbio, China) and detected by the BD FACSCalibur™ Flow Cytometer (BD Biosciences, USA).

### Network Pharmacological Analysis

The active ingredients of KTC were retrieved from the TCMSP database (https://old.tcmsp-e.com/tcmsp.php). Targets for active ingredients were then searched through the TCMSP database and the target names were converted to gene names through the uniprot database (https://www.uniprot.org/). Differentially expressed genes (DEGs) were screened in GEO database. Therapeutic targets obtained from CTD, DisGeNet, genecards, NCBI, OMIM, and PharmGKB databases were combined with genes from GEO databases to screen for targets that appeared in at least two databases. These targets were then crossed with KTC therapeutic targets to identify KTC therapeutic targets for PCOS. Finally, Cytoscape 3.7.2 software was used to construct the relationship network between KTC active components and target genes, and identify the active components related to the core target of KTC treatment of POF. Through cross-analysis of therapeutic targets of KTC and POF in bioinformatics database, potential therapeutic targets of KTC for POF were screened. PPI network was constructed using potential therapeutic targets, and core targets were further screened.

### Statistical Analysis

Data were presented as mean ± SD and analyzed using GraphPad PRISM 9.5.1 software. Differences were compared using ANOVA with the Bonferroni test. Statistical significance was defined as *P < 0.05, **P < 0.01, ***P < 0.001.

## Results

### KTC Improved Follicle Development in POF Rats

**Fig. 1:**
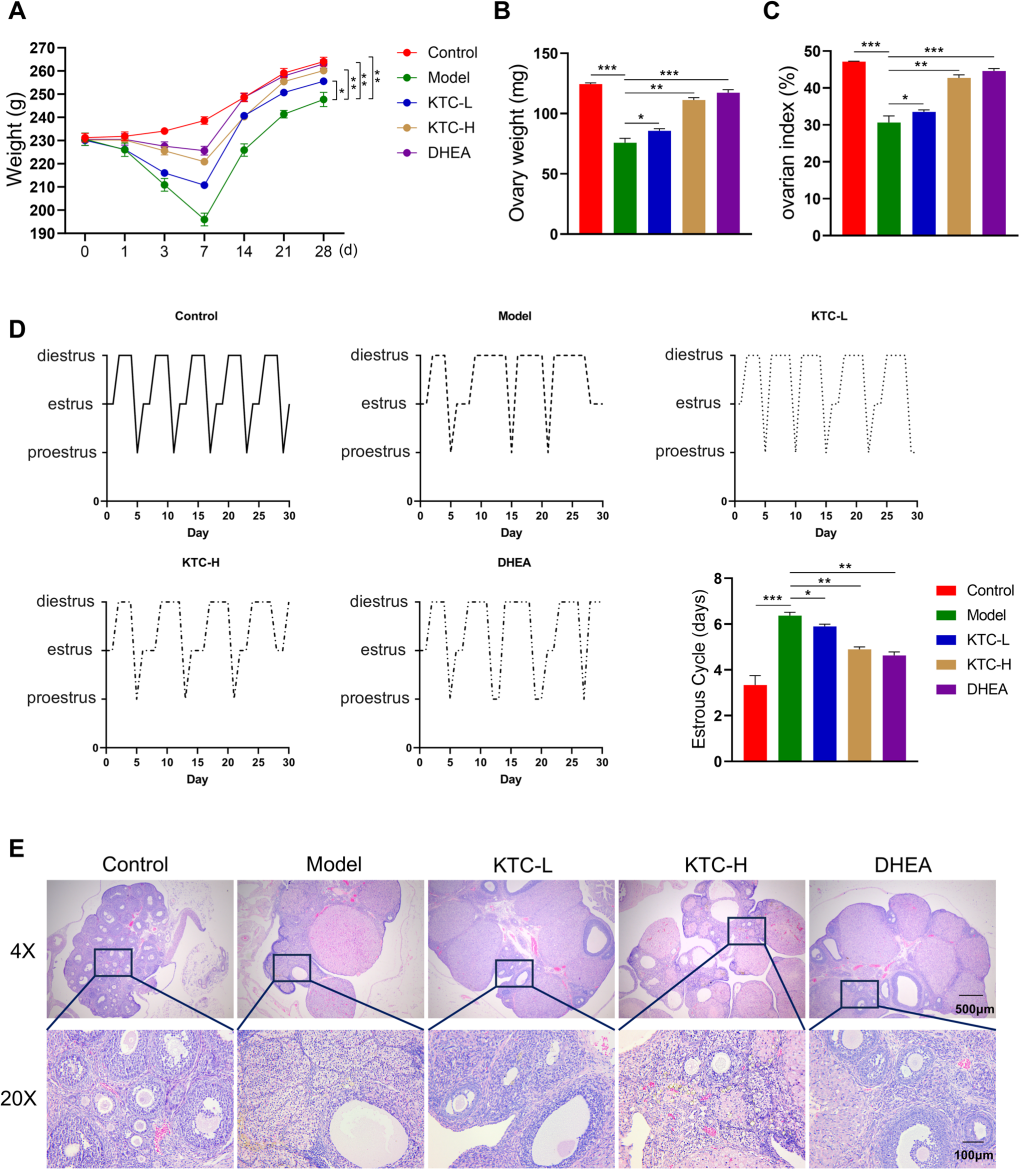
Effects of Kuntai capsules on the body weights and ovaries of rats. **A**: Curves of body weight changes in different groups of rats within 28 days; **B**: Ovary weights of the rats; **C**: Rat ovarian indices; **D**: Regular curves and average length histograms of the estrus cycles of rats of the rats in different groups; **E**: H&E staining of ovary sections from the different groups of rats. *P < 0.05, **P < 0.01, ***P < 0.001

In comparison to normal SD rats (Control group), POF rats across various groups exhibited varying degrees of weight loss from days 1 to 7; however, all groups began to regain weight after 14 days. When compared to untreated POF rats (Model group), POF rats treated with KTC or DHEA (DHEA group) experienced less weight loss by day 3 (Fig. 1A). At day 28, the rats were sacrificed and the ovarian tissues were removed. The ovarian weight in the Model group was significantly lower than that of the Control group (*P* < 0.001), while treatment with KTC or DHEA resulted in a significant increase in ovarian weight among POF rats (*P* < 0.05 vs. Model group) (Fig. 1B). The results pertaining to the ovarian index are illustrated in Fig. 1C. Additionally, there were notable differences in the average length and regularity of the estrus cycle between the Model and Control groups. Specifically, the average length of the estrus cycle was longer in the Model group compared to the Control group (*P* < 0.001), but it was shortened following treatment with KTC or DHEA (*P* < 0.05 vs. Model group) (Fig. 1D). Furthermore, when comparing low-concentration KTC treatment (KTC-L group) to high-concentration KTC treatment (KTC-H group), the changes in weight, ovarian weight, and average length of the estrus cycle in POF rats treated with high-concentration KTC were more analogous to those in the DHEA group.

The results of H&E staining are presented in Fig. 1E. In the Control group, there were more primordial follicles and more granular cell layers in mature follicles, arranged neatly. Conversely, in the Model group, the granulosa cells of mature follicles were fewer and disordered, there was a decreased number of primordial follicles, growing and mature follicles, and no corpus luteum formation was noted. In the KTC-L group, the arrangement of follicular granulosa cells was disordered, while the counts of primordial follicles, growing and mature follicles were higher than those in the Model group. The results of the KTC-H group and the DHEA group were comparable, as evidenced by the more organized arrangement of mature follicle granulosa cells. Furthermore, both groups exhibited a higher number of primordial follicles, growing and mature follicles compared to the Model group. Additionally, a small quantity of corpus luteum was observed.

**Fig. 2:**
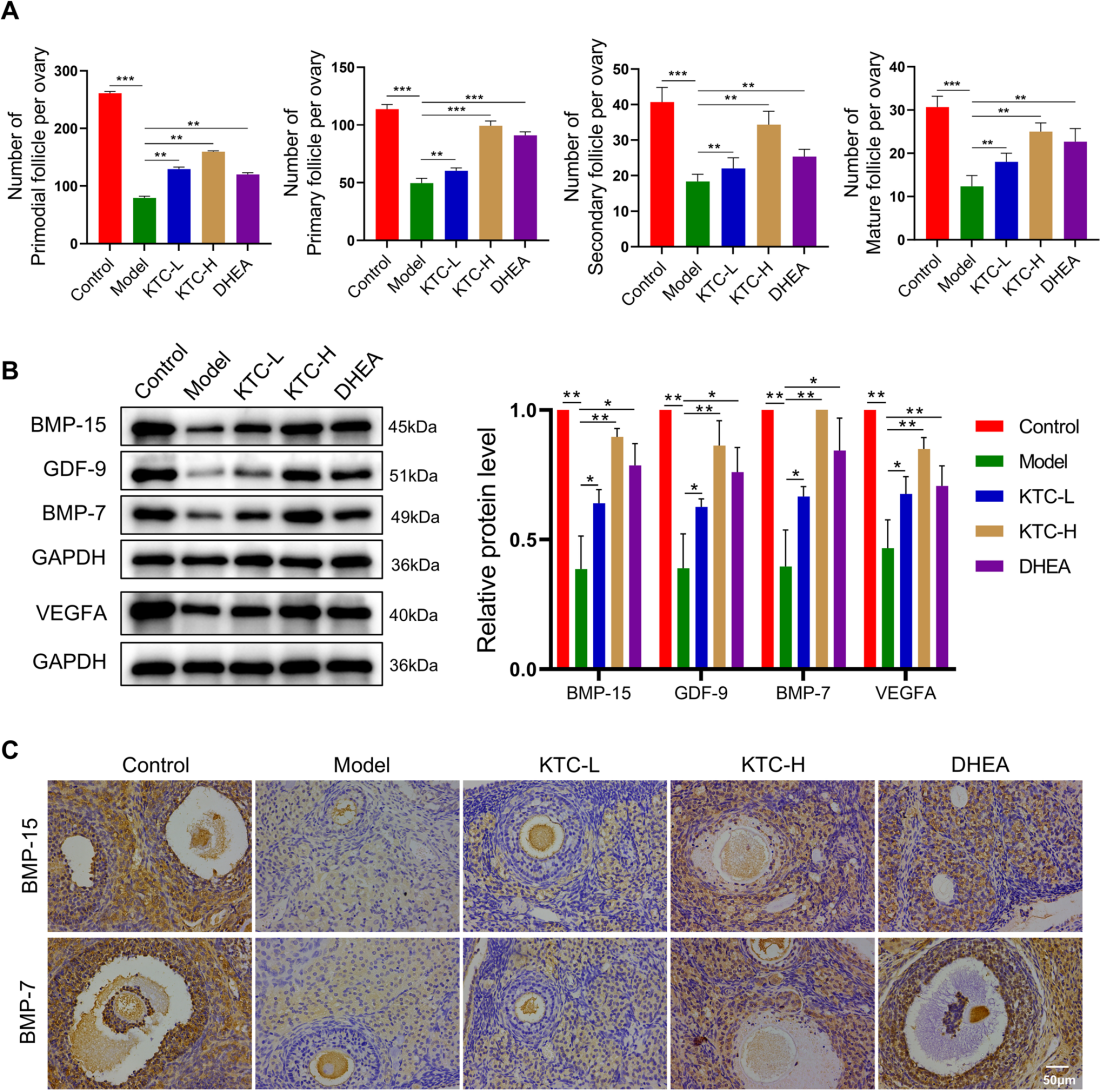
Effects of Kuntai capsules on follicle and blood vessel formation in rats. **A**: The number of follicles in the rats; **B**: Western blot results of rat ovarian growth-related proteins; **C**: Immunohistochemical images of the rat ovarian tissue sections. *P < 0.05, **P < 0.01, ***P < 0.001

The follicle count results are presented in Fig. 2A. The number of primordial follicles, primary follicles, secondary follicles, and mature follicles in the Model group was significantly lower than that in the Control group (*P* < 0.001). Notably, following treatment with KTC or DHEA, the KTC-H group exhibited the most pronounced increase in follicle count. Western blot (WB) analysis revealed that the expressions of BMP-15, GDF-9, BMP-7, and VEGFA were the lowest in the Model group compared to all other groups (*P* < 0.05). Additionally, the expression levels in the KTC-L group were lower than those in the KTC-H group, which, in turn, were slightly lower than those in the Control group (Fig. 2B). The results of immunohistochemistry corroborated the findings from the WB analysis. Specifically, the expressions of BMP-15 and BMP-7 in ovarian tissue were lowest in the Model group, highest in the Control group, and greater in the KTC-H group than in the KTC-L group (Fig. 2C).

## KTC Can Alleviate Premature Ovarian Failure and Ovarian Cell Apoptosis in POF Rats

**Fig. 3:**
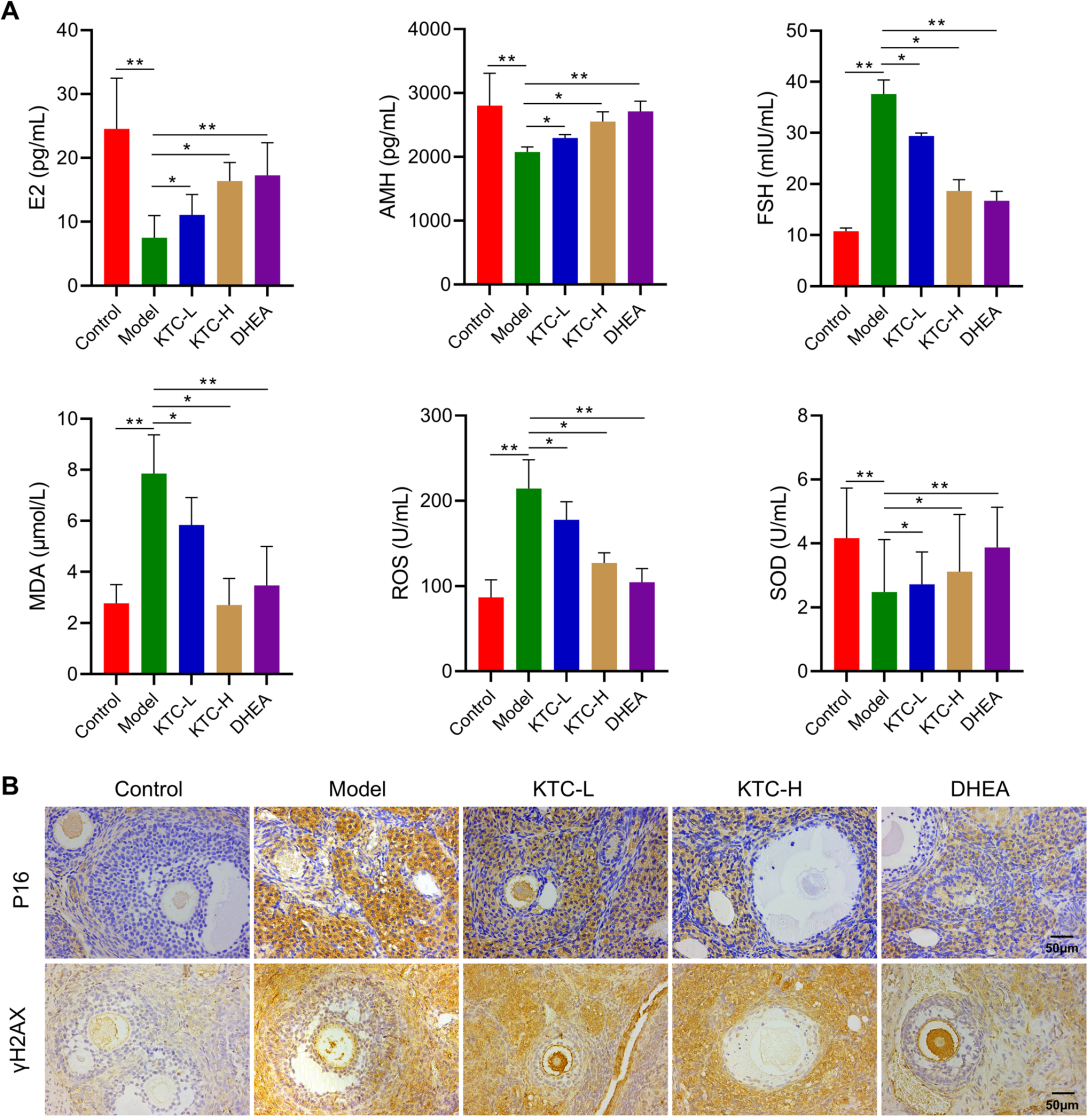
Effects of Kuntai capsules on hormone levels and aging in rat ovarian tissue. **A**: ELISA analysis of the serum hormone, oxidative metabolite and oxidase levels in the rats; **B**: Immunohistochemical map of the expression of markers of ovarian aging in the rats. *P < 0.05, **P < 0.01

The results of ELISA are presented in Fig. 3A. Compared to the Control group, the levels of E2, AMH, and SOD in the Model group were significantly decreased (*P* < 0.01), while the levels of FSH, MDA, and ROS were markedly increased (*P* < 0.01). In contrast, the levels of E2, AMH, and SOD in the KTC-L, KTC-H, and DHEA groups were higher than those observed in the Model group (*P* < 0.05 vs. Model group), whereas the levels of FSH, MDA, and ROS were lower than those in the Model group (*P* < 0.05 vs. Model group). Additionally, the immunohistochemical analysis indicated that the expression of P16 and γH2AX in the ovarian tissue of the Model group was elevated compared to the Control group. Furthermore, the levels of P16 and γH2AX in the ovarian tissue of the KTC-L, KTC-H, and DHEA groups were reduced compared to those in the Model group. Notably, the expression of P16 and γH2AX in ovarian granulosa cells and ovarian tissues of the KTC-H group was lower than that in the KTC-L group (Fig. 3B).

**Fig. 4:**
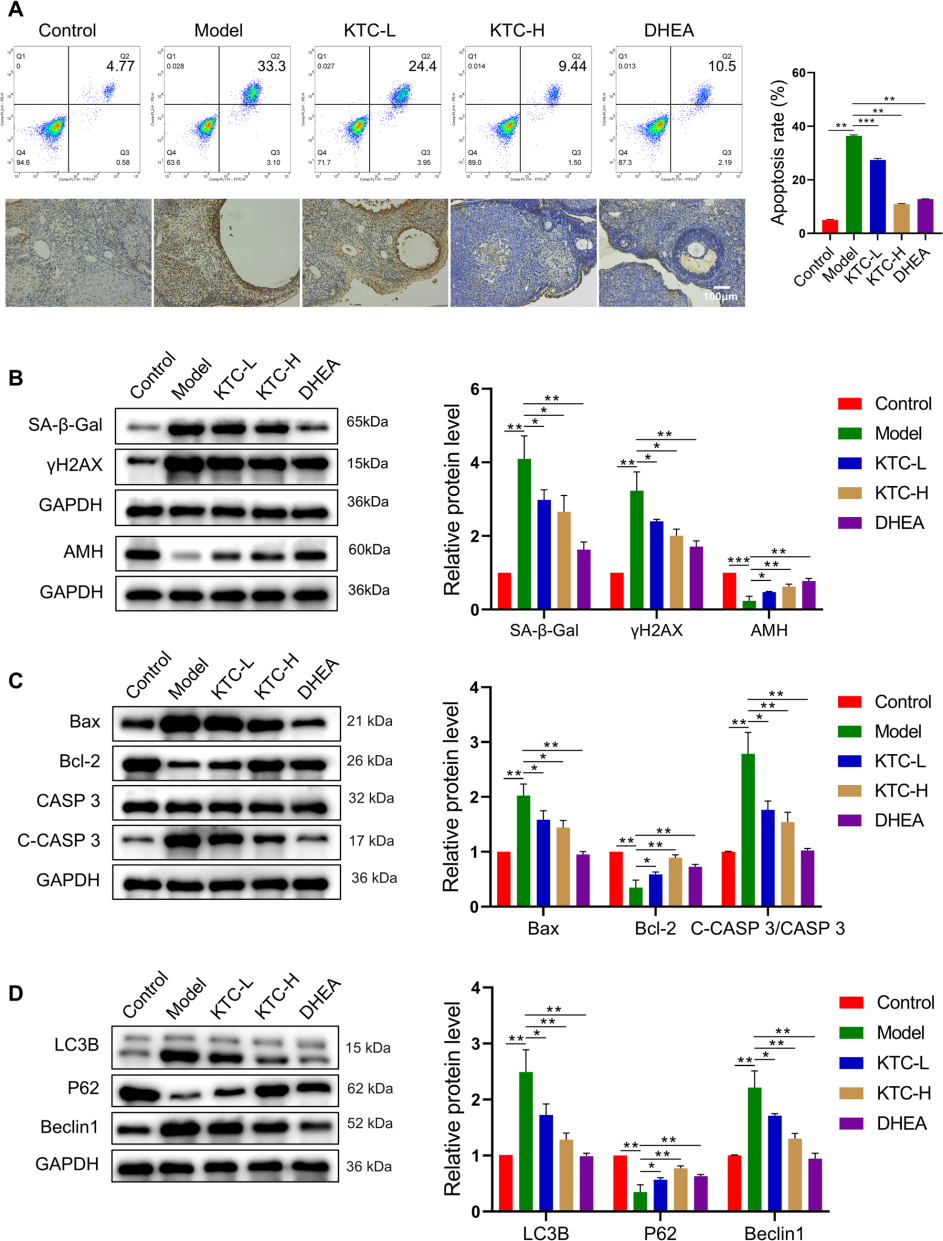
Effects of Kuntai capsules on ovarian apoptosis and autophagy in rats. **A**: Ovarian cell apoptosis was analyzed by flow cytometry and TUNEL staining. **B**: Relative expression of rat ovarian aging marker proteins. **C**: Relative expression of apoptosis marker proteins in rat ovarian tissue. **D**: Relative expression of autophagy marker proteins in rat ovarian tissue. *P < 0.05, **P < 0.01

Flow cytometry and TUNEL staining results, as illustrated in Fig. 4A, indicated that the apoptosis level of ovarian granulosa cells in the Model group was significantly higher than that in the Control group (*P* < 0.01). Furthermore, the apoptosis level of these cells decreased following treatment with KTC or DHEA (*P* < 0.01 vs. Model group). Notably, the apoptosis level in the KTC-H group was significantly lower than that in the KTC-L group (*P* < 0.001). WB analysis revealed that, compared to the Control group, the expression levels of SA-β-Gal, γH2AX, Bax, and Cleaved Caspase-3/Caspase-3 in ovarian granulosa cells of the Model group were elevated (*P* < 0.01), while the expressions of AMH and Bcl-2 were reduced (*P* < 0.01). In contrast, the expressions of SA-β-Gal, γH2AX, Bax, and Cleaved Caspase-3/Caspase-3 in the KTC-L, KTC-H, and DHEA groups were lower than those in the Model group (*P* < 0.05 vs. Model group), and the levels of AMH and Bcl-2 were higher than in the Model group (*P* < 0.05 vs. Model group) (Fig. 4B, 4C). Additionally, the expression of autophagy markers LC3B II/I and Beclin1 decreased with increasing KTC concentration (*P* < 0.05 vs. Model group), while the expression of P62 also showed a decreasing trend with higher KTC concentrations (*P* < 0.05 vs. Model group) (Fig. 4D).

## Network Pharmacology Predicted that KTC May Act Through AMPK-mTOR Pathway

**Fig. 5:**
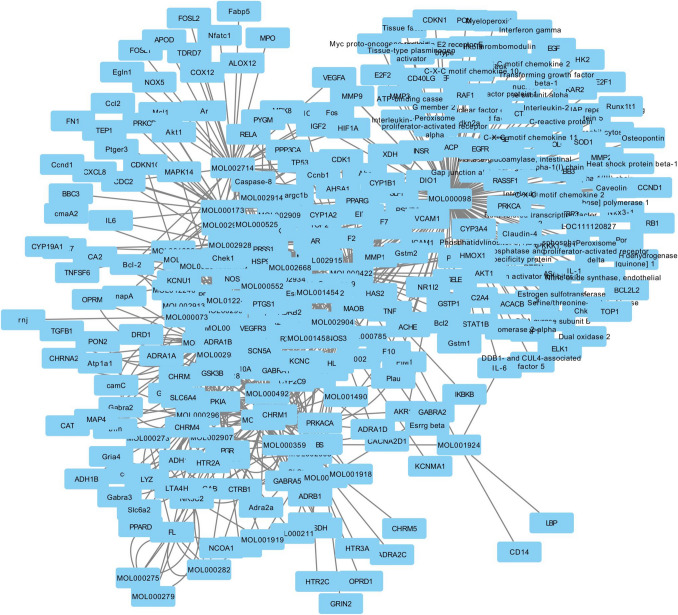
Network pharmacological enrichment analysis of Kuntai capsules

According to the screening criteria, the active components and therapeutic targets of KTC were identified using the TCMSP database. After converting the target names to gene names, the KTC regulatory network was constructed using Cytoscape software, as illustrated in the figure. The three primary medicinal components identified were quercetin, kaempferol, and luteolin. DEGs were observed in patients with POF compared to healthy individuals. Following the integration of DEGs with POF-related targets, potential therapeutic targets for POF were identified. Enrichment analysis revealed that these targets were primarily associated with the AMPK signaling pathway, IL-17 signaling pathway, and HIF-1 signaling pathway (Fig. 5).

## KTC Affects Autophagy and Alleviates POF through AMPK-mTOR Pathway

**Fig. 6:**
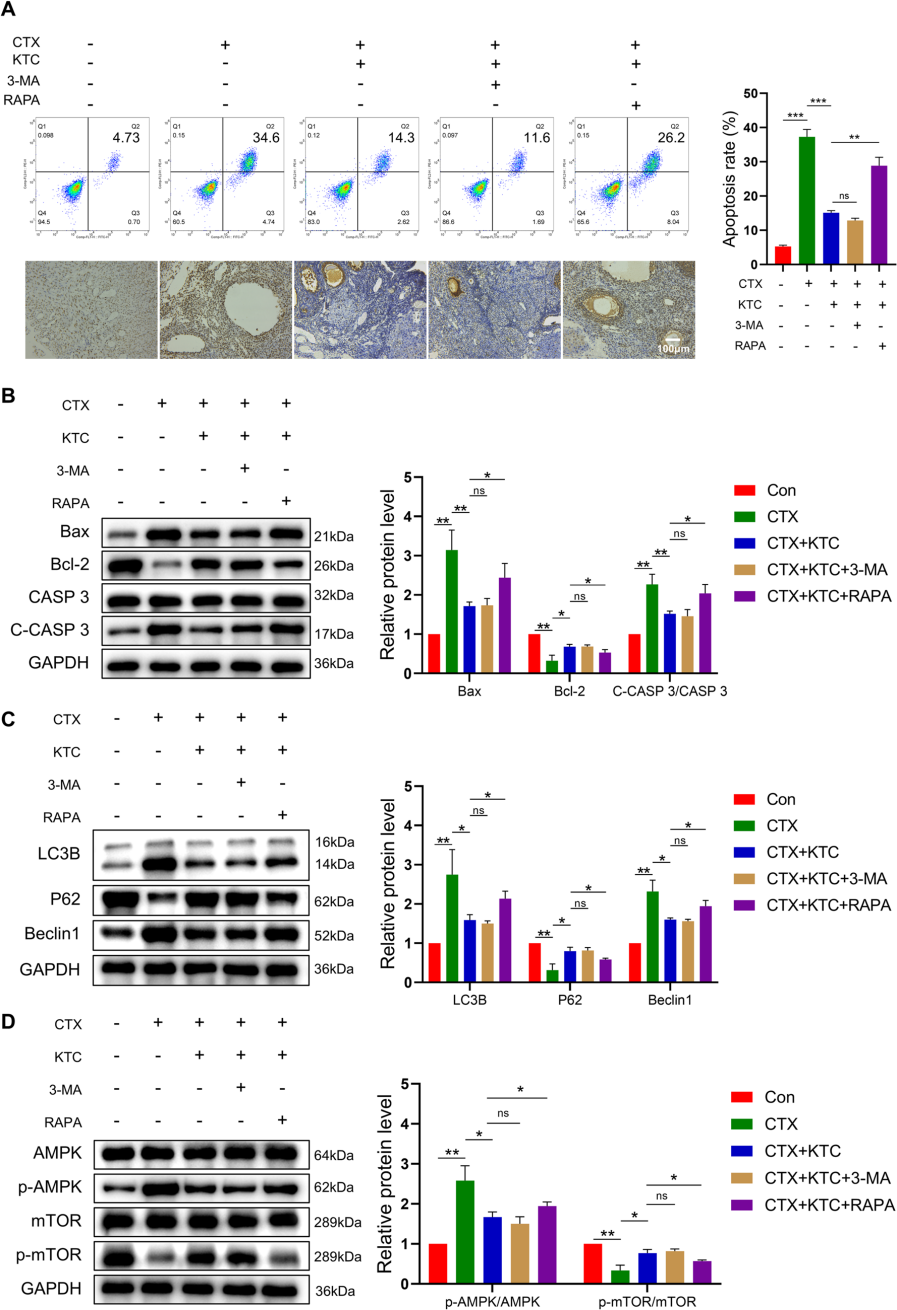
Kuntai capsules affect ovarian autophagy through the AMPK-mTOR pathway. **A**: Ovarian cell apoptosis was analyzed by flow cytometry and TUNEL staining. **B**: Relative expression of apoptosis marker proteins in rat ovarian tissue; **C**: Relative expression of autophagy marker proteins in rat ovarian tissue; **D**: Relative expression of AMPK‒mTOR pathway marker proteins in rat ovarian tissue. *P < 0.05, **P < 0.01, ***P < 0.001, ^ns^ *P* > 0.05

To validate whether KTC works through AMPK-mTOR signaling pathway, we administered a widely used inhibitor of autophagy, 3-Methyladenine (3-MA), and a specific mTOR inhibitor, rapamycin (RAPA) to the POF rats treated with KTC. Flow cytometry and TUNEL staining indicated that the level of apoptosis in POF rats was significantly higher than that in normal rats (*P* < 0.001), and that this level decreased following KTC treatment (*P* < 0.001). After KTC treatment and 3-MA treatment, no significant difference in the apoptosis level of POF rat ovarian cells was observed compared to the levels prior to 3-MA treatment. However, following treatment with KTC and the mTOR inhibitor RAPA, the level of apoptosis in ovarian cells was significantly higher than that without RAPA treatment (*P* < 0.01) (Fig. 6A). The Western blot results, as shown in Figs. 6B, 6C, and 6D, demonstrated that the expressions of Bax, Cleaved Caspase3/Caspase3, LC3B II/I, Beclin 1, and p-AMPK/AMPK were increased (*P* < 0.01), while the expressions of Bcl-2, P62, and p-mTOR/mTOR were decreased in POF rats (*P* < 0.01). After KTC treatment, the expressions of Bax, Cleaved Caspase3/Caspase3, LC3B II/I, Beclin 1, and p-AMPK/AMPK were reduced (*P* < 0.05 vs. CTX group), whereas the expressions of Bcl-2, P62, and p-mTOR/mTOR were elevated (*P* < 0.05 vs. CTX group). Compared to rats treated with KTC alone, there were no significant changes in the expression of these proteins following KTC treatment combined with 3-MA treatment. In contrast, after KTC treatment and RAPA treatment, the expressions of Bax, Cleaved Caspase3/Caspase3, LC3B II/I, Beclin 1, and p-AMPK/AMPK were increased (*P* < 0.05 vs. CTX + KTC group), while the expressions of Bcl-2, P62, and p-mTOR/mTOR were decreased (*P* < 0.05 vs. CTX + KTC group).

## Discussion

In recent years, Chinese proprietary medicine (CPM) has gained widespread use in the treatment of various diseases, owing to its advantages such as targeting multiple pathways and exhibiting few side effects [24]. Jia et al. demonstrated through systematic review and meta-analysis that CPM is more effective in improving osteoporosis and presents a lower risk of adverse events compared to standard Western medicine [25]. CPM has been shown to inhibit tumor occurrence and progression by targeting epithelial-mesenchymal transition (EMT), tumor angiogenesis, and other mechanisms [26]. Furthermore, CPM can enhance the therapeutic effects of antidepressants while reducing their side effects by regulating neurotransmitters and immune cell secretory factors [27]. Traditional CPMs are also employed in the treatment of ovarian-related diseases. The Kunling Pill can enhance the number and vitality of primordial follicles in patients with diminished ovarian reserve [28]. According to a Bayesian network meta-analysis conducted by Zhong et al., CPM combined with hormone replacement therapy (HRT) is more effective than HRT alone in treating primary ovarian insufficiency (POI), with KTC exhibiting the lowest incidence of adverse reactions [29]. Ma et al. further demonstrated through meta-analysis that KTC combined with Climen yields a higher overall treatment response rate in POF patients compared to Climen alone [30].

In our study, KTC demonstrated an improvement in the condition of small ovaries in POF rats. The estrus cycle of normal SD rats is categorized into four phases: early estrus, estrus, interestrus, and late estrus, which correspond to the early follicular, ovulation, luteal, and late luteal phases of the human menstrual cycle, respectively. Monitoring the estrus cycle of SD rats can provide insights into the effects of CTX-induced POF on the endocrine state and reproductive function of these animals [31]. Previous literature indicates that, compared to normal SD rats, the estrus cycle of POF rats is disrupted, specifically manifested as an extension in the length of the estrus cycle [32]. Furthermore, POF primarily arises through three mechanisms: accelerated follicular atresia, follicular dysplasia, and oocyte reduction [33]. Histological analysis in this study revealed that KTC ameliorated follicular atresia and positively influenced the number and arrangement of follicular granulosa cells in POF rats. Additionally, POF can result in decreased ovarian reserve function, with follicle count serving as a key indicator of this reserve [34]. The development of follicles can be divided into four stages: primordial, primary, secondary, and mature follicles. The primordial follicle pool is a critical determinant of ovarian reserve and reproductive lifespan [35]. The loss of primordial follicles is considered an early indicator of ovarian decline. In our study, we observed a significant reduction in the number of primordial follicles in CTX treated rats. Treatment with KTC resulted in an increase in the number of primordial follicles in POF rats. This effect was more pronounced with higher doses of KTC, suggesting that KTC may have a protective or restorative effect on the primordial follicle pool. A reduction in primary follicles may signify a diminished ovarian response to hormonal stimulation like FSH [36]. FSH is a critical hormone that binds to its receptors on granulosa cells, stimulating their proliferation and differentiation, which is essential for the progression of primary follicles to the secondary stage. A decrease in secondary follicles is linked to ovarian insufficiency, and mature follicles are associated with ovulation disorders and reduced fertility [37]. In this study, besides the primordial follicles, the number of primary, secondary and mature follicles also decreased following CTX treatment; however, following KTC treatment, the number of these follicles increased. These findings align with previous literature, suggesting that KTC enhances follicle development in POF rats [10]. Notably, a higher dose of KTC was found to be more effective than a lower dose in treating POF rats. The ability of KTC to positively influence the follicle pool indicates its potential as a therapeutic agent for conditions such as POF, where ovarian reserve is severely compromised.

SA-β-Gal and γH2AX are commonly employed to evaluate the extent of ovarian cell senescence in POF [38]. Following KTC treatment, the expressions of SA-β-Gal and γH2AX in ovarian granulosa cells and tissue sections from POF rats were found to be decreased, suggesting that KTC may ameliorate POF. The condition of granulosa cells serves as a critical indicator of oocyte quality, as accelerated follicular atresia fundamentally represents heightened apoptosis of granulosa cells [39]. The expressions of Bax, Bcl-2, and Caspase-3 are closely associated with the apoptosis of granulosa cells. In instances of POF, the expressions of Bax and Cleaved Caspase-3/Caspase-3 are elevated, while the expression of Bcl-2 is reduced, leading to an increased percentage of apoptosis [40]. After KTC treatment, the expression levels of Bax and Cleaved Caspase-3/Caspase-3 in the granulosa cells of POF rats decreased, while Bcl-2 expression increased, indicating a reduction in granulosa cell apoptosis. Ovarian follicles and the corpus luteum contain endothelium-specific factors that regulate angiogenesis, either independently or synergistically. Dysregulated expression of these factors can lead to angiogenesis disorders and the progression of ovarian diseases [41]. BMP-15, GDF-9, and BMP-7 are growth factors closely linked to follicle development. BMP-15 promotes the proliferation of granulosa cells, GDF-9 is specifically expressed in oocytes and plays a crucial role in the growth and maturation of follicles, while BMP-7 influences the proliferation and differentiation of granulosa cells [42]. Angiogenesis is vital for follicle growth and maturation, as blood vessels supply essential nutrients to the follicles; an adequate blood supply is a prerequisite for the normal development of follicles [43]. VEGFA and CD31 were employed to assess angiogenesis. Following KTC treatment, the expressions of BMP-15, GDF-9, BMP-7, VEGFA, and CD31 were found to be elevated, indicating that KTC may enhance follicular dysplasia and counteract the reduced ovarian angiogenesis associated with POF.

With the widespread application of CPM, it is crucial to identify the active ingredients and targets of drugs to enhance CPM. In this study, the therapeutic targets of KTC exhibited significant overlap with those associated with POF, with the AMPK/mTOR pathway showing the highest recurrence rate. The AMPK/mTOR signaling pathway is implicated in the development and activation of follicles; activation of AMPK can promote oocyte maturation, although it has also been reported that AMPK signaling activation may inhibit the mitosis of granulosa cells, thereby affecting follicular development [44]. An imbalance in autophagy levels can lead to impaired viability of granulosa cells and hinder the oogonia-to-oocyte transformation, which is a critical mechanism in the pathogenesis of POF [45]. The transformation of oogonia into oocytes is a complex process that involves meiosis and the formation of primordial follicles. Autophagy plays a crucial role in maintaining the health and viability of oocytes during this transformation by removing damaged organelles and proteins. Disruptions in autophagy can result in increased apoptosis and follicle atresia, ultimately reducing the ovarian reserve and contributing to POF. The AMPK/mTOR signaling pathway is closely linked to autophagy. Dai et al. utilized human umbilical cord-derived mesenchymal stem cells (hUC-MSCs) to enhance ovarian function in POF rats, finding that hUC-MSCs exert their effects by reducing oxidative stress and inhibiting excessive autophagy in ovarian granulosa cells via the PI3K/AKT/mTOR pathway [46]. In this study, KTC treatment activated the AMPK/mTOR pathway and resulted in decreased autophagy levels. It is likely that KTC inhibits autophagy by activating the AMPK/mTOR pathway, thereby contributing to improved follicle development and function in POF rats.

In conclusion, this study shows that KTC may inhibit autophagy by activating AMPK/mTOR pathway, thus playing a certain curative effect on POF. This provides a new theoretical basis for the treatment of POF by KTC.

## Supplementary Information

Below is the link to the electronic supplementary material.

Supplementary file1 (PDF 379 KB)

## Data Availability

The data used and/or analyzed during the current study are available from the corresponding author on reasonable request.

## Abbreviations

3-MA: 3-Methyladenine
AMH: Anti-Müllerian hormone
AMPK: Adenosine 5’-monophosphate (AMP)-activated protein kinase
BMP-15: Bone morphogenetic protein-15
BMP-7: Bone morphogenetic protein-7
CPM: Chinese proprietary medicine
CTX: Cyclophosphamide
DHEA: Dehydroisoandrosterone
E2: Estradiol
FSH: Follicle-stimulating hormone
GDF-9: Growth differentiation factor 9
H&E: Hematoxylin and eosin
hUC-MSCs: Human umbilical cord-derived mesenchymal stem cells
IL-17: Interleukin 17
KTC: Kuntai capsule
LC3B: Microtubule-associated protein 1 light chain 3 B
MDA: Malondialdehyde
mTOR: Mammalian target of rapamycin
P16: Multiple tumor suppressor 1
P62: Sequestosome 1
POF: Premature ovarian failure
RAPA: Rapamycin
ROS: Reactive oxygen species
SOD: Superoxide dismutase
TUNEL: Terminal deoxynucleotidyl transferase-mediated dUTP nick-end labeling
VEGFA: Vascular endothelial growth factor A
γH2AX: Phosphorylated histone H2AX
