# Supplementary figures and images for: Kuntai Capsules Improve Premature Ovarian Failure by Regulating AMPK-Mediated Autophagy
Source: Reprod Sci. 2025 Aug 11;32(9):2970–84. doi: 10.1007/s43032-025-01949-w (PMC12443895; doi:10.1007/s43032-025-01949-w)

Figure 2B

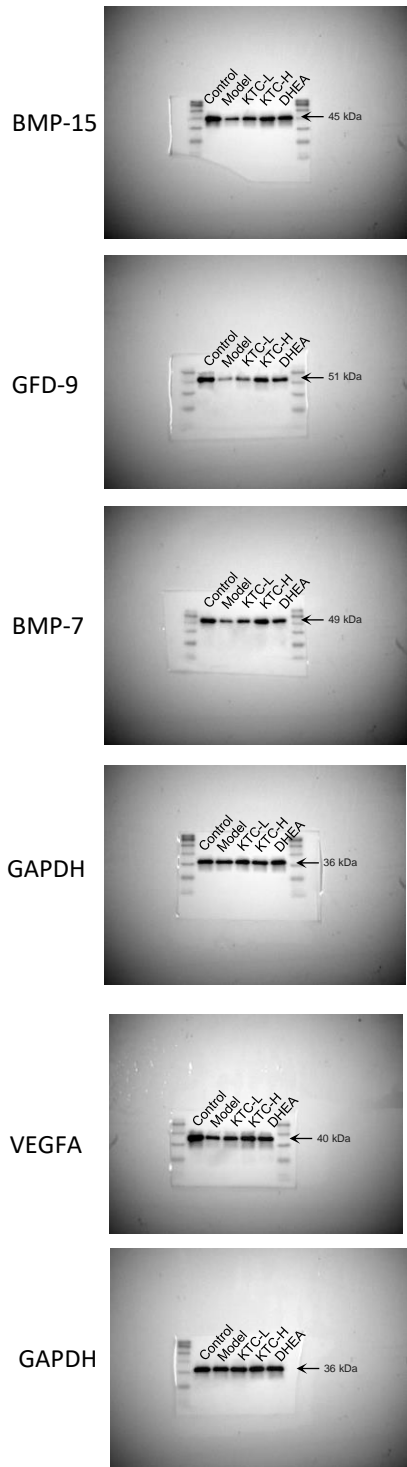

Figure 4B

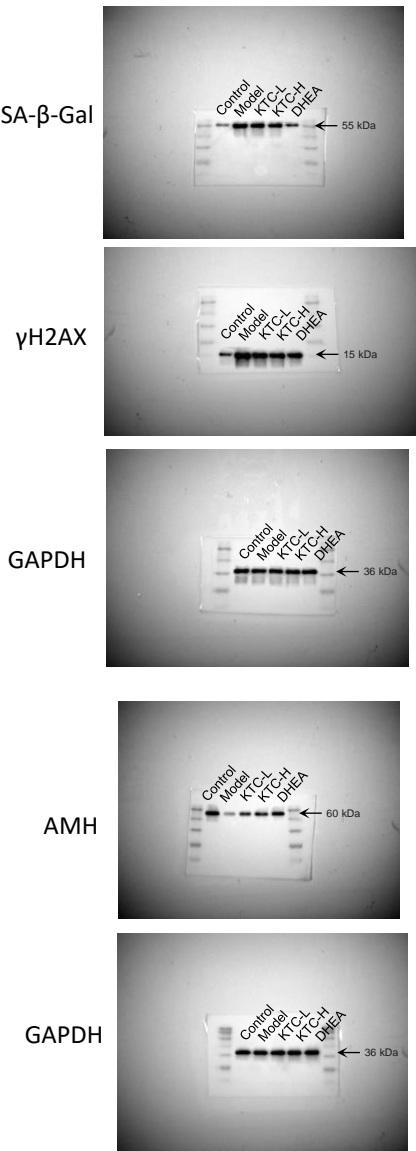

Figure 4C

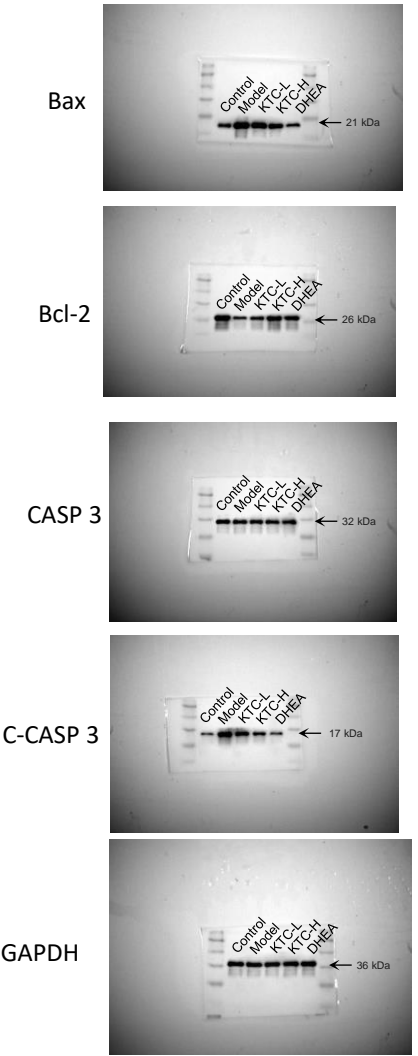

Figure 4D

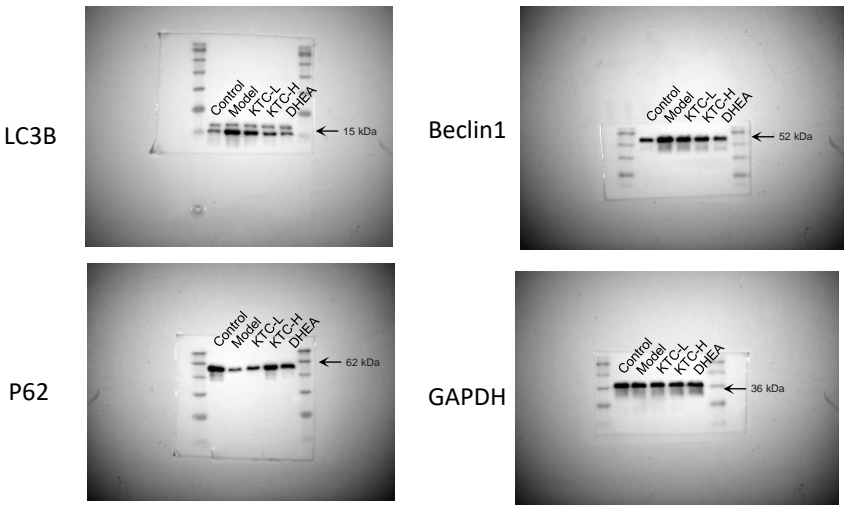

Figure 6B

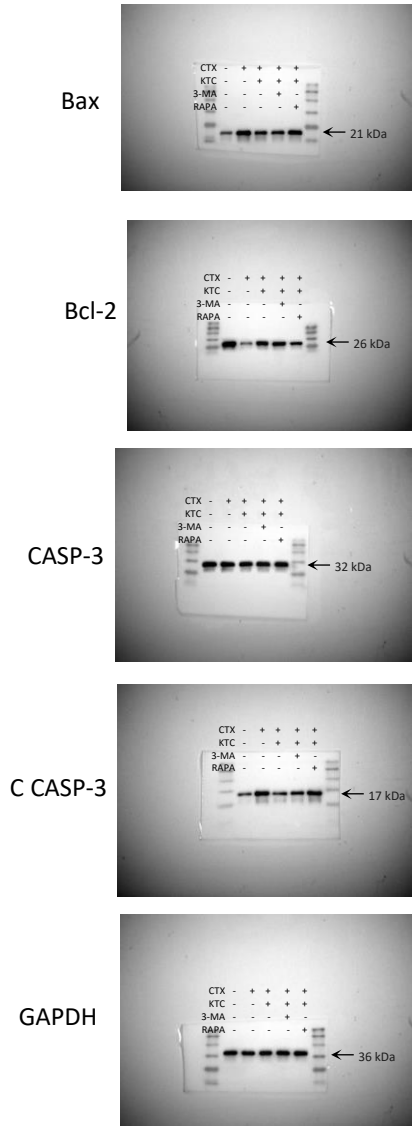

Figure 6C

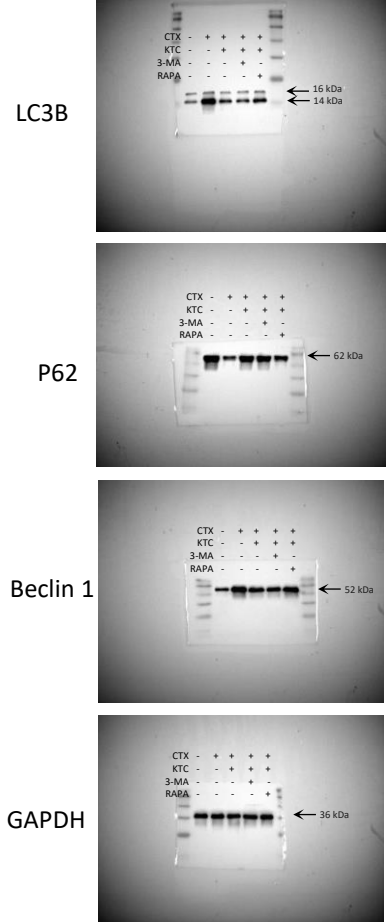

Figure 6D

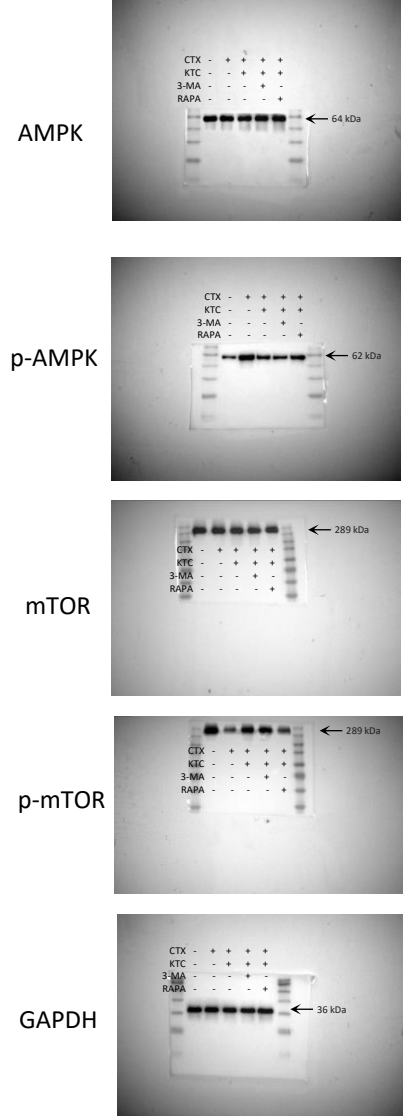

Supplement: Supplementary file 1 — Supplementary file1 (PDF 379 KB) [file 43032_2025_1949_MOESM1_ESM.pdf]
